# A high centrifugal force-enhanced Ziehl–Neelsen method for improved detection of *Mycobacterium tuberculosis*

**DOI:** 10.1371/journal.pone.0342273

**Published:** 2026-07-02

**Authors:** Godlove T. Chaula, Lucy Namkinga, Wilber Sabiiti, Nyanda E. Ntiningya, Bariki Mtafya, Ally Mahadhy

**Affiliations:** 1 Mbeya Medical Research Centre, National Institute for Medical Research, Mbeya, Tanzania; 2 Department of Biochemistry and Pharmacology, Mbeya Collage of Health and Allied Sciences, University of Dar es Salaam, Mbeya, Tanzania; 3 School of Medicine, University of St Andrews, St. Andrews, United Kingdom; 4 Department of Molecular Biology and Biotechnology, College of Natural and Applied Sciences, University of Dar es Salaam, Dar es salaam, Tanzania; Hangzhou Red Cross Hospital, CHINA

## Abstract

Ziehl–Neelsen (ZN) smear microscopy remains central to tuberculosis (TB) diagnosis and treatment monitoring; however, its sensitivity is limited by incomplete recovery of *Mycobacterium tuberculosis* during pre-analytical processing. This study evaluated whether modifying centrifugation force and duration improves bacillary recovery and ZN smear microscopy performance. Laboratory experiments were conducted using *M. tuberculosis* H37Rv suspensions and clinical sputum specimens. Following NALC–NaOH treatment, samples were centrifuged at 2,000, 3,000, and 6,000 × g for 40 min. The effect of centrifugation duration was assessed at 3,000 × g by comparing 20 and 40 min using the same *M. tuberculosis* H37Rv cultures and the same clinical sputum specimens at both time points, ensuring paired measurements within each sample type. Smear positivity and ZN smear grading were evaluated from replicate smears and analyzed using non-parametric statistical tests, with significance set at *p* < 0.05. In *M. tuberculosis* H37Rv suspensions, no significant differences in smear positivity or grading were observed across centrifugal forces (*p* = 0.368 and *p* = 0.212, respectively). In clinical sputum specimens, smear positivity did not differ significantly across forces (*p* = 0.716), whereas ZN smear grading increased significantly with higher centrifugal force (*p* = 0.0051). At 3,000 × g, extending centrifugation time from 20 to 40 min did not significantly affect smear positivity in either sample type (both *p* = 1.000). In contrast, ZN smear grading increased from 1+ to 2+ in clinical specimens with extended centrifugation time (*p* = 0.016), while no change was observed in *M. tuberculosis* H37Rv suspensions. These findings indicate that increasing centrifugal force may enhance bacillary concentration in clinical sputum, resulting in improved smear grading without a corresponding increase in detection rate. Extending centrifugation time has limited impact on smear positivity. Optimization of pre-analytical centrifugation parameters may improve ZN smear microscopy performance in clinical specimens.

## Introduction

Tuberculosis (TB) remains a major global public health challenge, with an estimated 10 million new cases and over 1 million deaths reported annually [1]. Controlling TB requires not only effective diagnostics and treatment but also innovative tools to monitor treatment response. Despite the availability of effective treatment regimens, TB elimination has yet to be achieved, largely due to persistent diagnostic gaps. In 2022, approximately 30% of individuals with TB were not diagnosed and, consequently, did not receive treatment [2]. Moreover, 37% of diagnosed cases were based solely on clinical assessment, which often lacks sufficient specificity and sensitivity [3,4]. These limitations highlight the need to optimize the use of existing diagnostic tools to improve case detection and reduce missed diagnoses.

The prolonged duration of tuberculosis (TB) treatment, combined with multi-drug regimens that may be toxic, not only increases costs but also poses significant safety concerns for patients [5]. The situation is further complicated by the emergence of drug-resistant TB (DR-TB), which requires more complex, toxic, and prolonged treatment compared to drug-susceptible TB (DS-TB). This underscores the critical need for effective tools to monitor treatment response [6], enabling early identification of patients who are not responding adequately to therapy or who are at risk of relapse or treatment failure [7,8].

Currently, Ziehl–Neelsen (ZN) smear microscopy remains a standard diagnostic method for TB [9]. In addition, clinical sputum specimens are commonly processed using the N-acetyl-L-cysteine (NALC)–sodium hydroxide (NaOH) method followed by standard centrifugation at 3000 × g for 15–20 minutes prior to ZN smear microscopy [10,11]. However, this approach has several technical limitations, including low sensitivity due to poor recovery of bacilli during centrifugation [12,13].

Given the low specific gravity of *Mycobacterium tuberculosis* (ranging from 1.07 to 0.79), a high relative centrifugal force (RCF) is necessary to effectively concentrate the bacilli into the pellet [14]. However, previous studies have shown that standard centrifugation settings of 3000–3500 × g for 20 minutes may result in more than 90% of mycobacterial cells remaining unrecovered in the supernatant [15]. Therefore, there is a need to re-evaluate the current standard centrifugation protocol of 3000 × g for 20 minutes to improve recovery of *M. tuberculosis* for ZN smear microscopy. In this context, we examined the effect of increasing centrifugal force at a fixed centrifugation duration on the recovery of *M. tuberculosis* cells for ZN smear microscopy.

## Materials and methods

### Study samples

In this study, *M. tuberculosis* H37Rv (ATCC 27294) laboratory strain and clinical pulmonary tuberculosis sputum samples were used to assess the impact of relative centrifugal force at a fixed duration on the recovery of *M. tuberculosis*, followed by ZN smear microscopy. Clinical sputum samples were obtained from adult participants aged 18–65 years who were recruited at Rungwe District Hospital between 30/07/2020 and 29/07/2021 as part of the TB-MBLA translational study. A total of 10 pooled clinical sputum specimens were included, each prepared by combining spot and early morning sputum samples collected from microbiologically confirmed TB-positive participants (n = 10). Bacillary burden, as determined by routine smear microscopy performed at the originating diagnostic laboratory, ranged from scanty to 3 + smear grades according to standard ZN microscopy criteria. Microbiological status was previously confirmed using the GeneXpert MTB/RIF assay. Clinical and laboratory data were accessed for research purposes on a rolling basis between 30/06/2021 and 29/07/2021.

### Ethics statement

This study was nested within the TB-MBLA translational study conducted under routine healthcare practice in Mbeya, Tanzania. Ethical approval was obtained from the Mbeya Medical Research and Ethics Committee (SCEC-2439/R. E/V.1/82) and the Medical Ethics and Research Coordinating Committee of the National Institute for Medical Research (NIMR/HQ/R.8a/Vol. IX/3687), in accordance with national research ethics guidelines. Written informed consent was obtained from all participants prior to enrolment. For participants who were unable to read or write, the study information and consent form were read aloud in the local language (Swahili), and verbal informed consent was obtained in the presence of an independent witness. Consent was documented by a thumbprint and witness signature on the consent form. The verbal consent procedure, including its documentation process, was reviewed and approved by the Institutional Review Board (IRB)/ethics committee.

### Ziehl–Neelsen smear grading and positivity

The effect of relative centrifugal force (RCF) on the recovery of *M. tuberculosis* from samples was assessed using ZN smear microscopy, with both smear grading and positivity determined as previously described [14]. Following acid-fast staining, bacilli were observed and counted under a microscope. Smears were graded according to the number of acid-fast bacilli (AFB) observed: if no AFB were detected in 100 fields, the result was considered negative; 1–9 AFB in 100 fields was classified as scanty; 10–99 AFB in 100 fields was graded as 1 + ; 1–10 AFB per field in at least 50 fields was graded as 2 + ; and more than 10 AFB per field in at least 20 fields was graded as 3 + .

The primary microscopist was not blinded to experimental conditions. A subset of slides showing low or uncertain grading was independently re-read by a second microscopist who was blinded to processing conditions to reduce classification uncertainty. Formal inter-reader variability analysis was not formally performed because secondary review was limited to uncertain slides; however, the available readings showed general agreement in smear interpretation.

ZN smear positivity (%) was then calculated using the formula [16]:


$$\begin{array}{c} SmearPositivity(\%)=\frac{Numberofpositivesmears}{Totalnumberofsmearsexamined}\times \text{1}\text{0}\text{0} \end{array}$$ (1)

### Effect of centrifugal force on recovery of M. tuberculosis H37Rv

*In vitro* experiments were performed using a 2-week-old culture of *M. tuberculosis* H37Rv strain (ATCC 27294) grown on Löwenstein–Jensen (LJ) medium. Three loopfuls of pure colonies were suspended in Middlebrook 7H9 broth. The suspension was adjusted to 0.5 McFarland standard (approximately 1.0 × 10⁸ CFU/mL) and serially diluted in Middlebrook 7H9 broth to a final concentration of 100 CFU/mL. An equal volume of NALC–NaOH solution (1% N-acetyl-L-cysteine and 2% sodium hydroxide) was added and incubated for 20 min. The processed suspension was centrifuged at 2000 × g, 3000 × g, and 6000 × g for 40 min at 4 °C [17]. Each relative centrifugal force (RCF) condition was evaluated in five independent experimental replicates conducted on separate culture preparations (n = 5). Each replicate was processed independently through the full workflow, and one smear was prepared for microscopic analysis per replicate. Smears were stained using the ZN method, examined microscopically, and graded according to WHO guidelines [16].

### Effect of centrifugal force on recovery of M. tuberculosis in clinical sputum samples

Ten pooled clinical sputum specimens (n = 10) were processed using the NALC–NaOH method as described below. Each specimen was homogenized using a sterile magnetic stirrer for 20 min at room temperature. From each specimen, 2 mL of sputum was subjected to decontamination using NALC–NaOH for 20 min. The resulting suspension was centrifuged at 2000 × g, 3000 × g, and 6000 × g for 40 min at 4 °C [17]. Pellets were resuspended in 2 mL phosphate-buffered saline (PBS), and smears were prepared using 50 µL aliquots. Smears were stained using the Ziehl–Neelsen (ZN) method, examined microscopically, and graded according to WHO guidelines [16].

### Quality control and replicates

For the *M. tuberculosis* H37Rv laboratory strain, each relative centrifugal force (RCF) condition was evaluated in five independent experimental replicates conducted on separate culture preparations. For clinical sputum specimens, ten pooled specimens were included, and each specimen was processed under each RCF condition, from which three technical replicate smears were prepared. A control condition of 3,000 × g for 20 min was included to account for baseline processing effects. All centrifugation steps were performed at 4 °C, and both the centrifuge and microscope were calibrated prior to each experiment to ensure consistency and reproducibility. For statistical analysis, results from technical replicate smears were summarized at the specimen level, and these aggregated values were used as the unit of analysis.

### Data analysis

Data were entered into Microsoft Excel and analyzed using GraphPad Prism (GraphPad Software, La Jolla, CA, USA). The unit of analysis was the independent experimental replicate for Mycobacterium tuberculosis H37Rv experiments (n = 5) and the clinical sputum specimen for clinical sample experiments (n = 10). For both experimental systems, three technical replicate smears were prepared per unit under each experimental condition. Smear results were aggregated at the level of the independent experimental unit. A unit was classified as positive if at least one of the three smears was positive, and the final ZN grade was assigned as the modal grade across the three smears. Technical replicates were used for measurement reliability only and were not treated as independent observations in the analysis.

For statistical analysis, ZN grades (scanty, 1 + , 2 + , and 3+) were treated as ordinal variables reflecting increasing bacillary load. The median score per unit was used for analysis. Smear positivity was derived from the same unit-level data and expressed as a binary outcome (positive or negative) under each experimental condition.

Comparisons of ZN smear grading across centrifugal force conditions (2,000 × g, 3,000 × g, and 6,000 × g for 40 min) were performed using the Friedman test. Differences in paired smear positivity across centrifugal force conditions were analyzed using Cochran’s Q test. For centrifugation time comparisons (3,000 × g for 20 min versus 40 min), McNemar’s test was used for smear positivity, while the Wilcoxon signed-rank test was used for ZN grading.

Effect sizes for smear positivity were estimated as paired proportion differences with corresponding 95% confidence intervals (95% CI). All tests were two-sided, and statistical significance was set at *p* < 0.05. Non-parametric tests were used because ZN grading data were ordinal and did not meet assumptions of normality.

## Results and discussion

### Recovery of M. tuberculosis H37Rv at different RCF (ZN microscopy)

**Fig 1:**
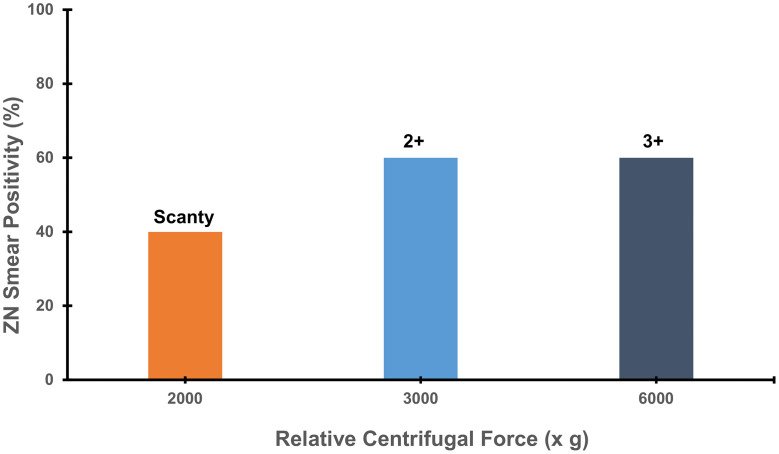
Smear positivity (%) and median ZN smear grade of *M. tuberculosis* H37Rv samples processed at different relative centrifugal forces (RCFs). Bars represent the percentage of positive smears (n = 5 independent experimental replicates per condition). Values above bars indicate the median ZN smear grade for each condition.

*M. tuberculosis* H37Rv suspensions were centrifuged at 2,000, 3,000, and 6,000 × g, and ZN smear positivity and grading were assessed (Fig 1).

Smear positivity increased from 40% (95% CI: 11.8–77.0%) at 2,000 × g to 60% (95% CI: 23.1–88.3%) at 3,000 × g and remained unchanged at 60% (95% CI: 23.1–88.3%) at 6,000 × g; however, these differences were not statistically significant (*p* = 0.368). Similarly, smear grading showed an increasing trend with higher relative centrifugal force, although this did not reach statistical significance (*p* = 0.212). The lack of statistical significance may be related to the limited number of experimental replicates (n = 5 per condition) and variability in bacillary distribution within the suspension.

Overall, increasing relative centrifugal force was associated with higher smear positivity from at 3,000 × g, followed by a plateau at 6,000 × g. Variability in positivity across replicates likely reflects heterogeneity in bacillary distribution within the suspension. At 2,000 × g, incomplete sedimentation may reduce bacillary recovery and lead to occasional false-negative smears, whereas at 3,000 × g, improved sedimentation reduces this variability and increases detection consistency. Further increases in RCF did not materially affect positivity. Although smear positivity plateaued at higher centrifugal force, smear grading continued to increase at 6,000 × g, indicating a higher bacillary load per microscopic field. This suggests that increasing centrifugal force enhances quantitative bacillary concentration even when detection rates remain unchanged.

To further examine the influence of centrifugation time, samples were processed at 3,000 × g for 20 and 40 min. Smear positivity and grading remained unchanged between durations, with 60% positivity (95% CI: 17–100%) and a median ZN grade of 2 + observed under both conditions. No statistically significant differences were detected between the two centrifugation durations (both *p* = 1.000). These results suggest that most detectable bacilli from *M. tuberculosis* H37Rv suspensions are recovered within the first 20 min, with only a marginal gain in recovery achieved by extending centrifugation time.

Taken together, 3,000 × g provides sufficient centrifugal force to achieve maximal smear positivity under the conditions tested, while higher forces may further increase bacillary concentration without improving ZN smear microscopy performance.

### Recovery of *M. tuberculosis* from sputum at different RCF (ZN microscopy)

**Fig 2:**
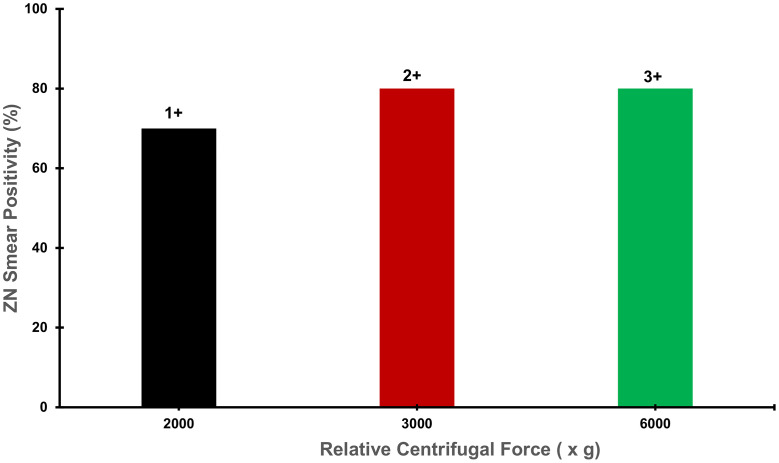
Smear positivity (%) and median ZN smear grade of *M. tuberculosis* in clinical sputum samples processed at different relative centrifugal forces (RCFs). Bars represent the percentage of positive smears (n = 10 clinical sputum specimens, analyzed under each condition). Values above bars indicate the median ZN smear grade for each condition.

The efficiency of *M. tuberculosis* recovery from clinical sputum samples was evaluated at different relative centrifugal forces (2,000, 3,000, and 6,000 × g) using ZN smear microscopy and grading (Fig 2).

In contrast to the *M. tuberculosis* H37Rv laboratory strain, clinical sputum specimens showed higher smear grades and an overall increasing trend in positivity with increasing relative centrifugal force (RCF). At 2,000 × g, incomplete sedimentation may have reduced bacillary recovery, resulting in lower smear positivity (95% CI: 39.7–89.2%) and lower smear grades. Increasing the RCF to 3,000 × g was associated with improved smear positivity, while 6,000 × g showed a further increase in bacillary intensity, reflecting enhanced concentration of bacilli within the sputum matrix. The difference in positivity across the three conditions was not statistically significant (*p* = 0.716), with smear positivity reaching 80% at both 3,000 × g and 6,000 × g (95% CI: 49.0–94.3%). In contrast, ZN smear grading increased significantly with increasing RCF (*p* = 0.0051).

These findings highlight the influence of specimen complexity on centrifugation performance. Clinical sputum contains mucus, host cells, and debris that may hinder bacillary sedimentation and distribution [14]. In contrast, the laboratory strain showed a plateau in positivity at 3,000 × g, suggesting that optimal centrifugation parameters differ between standardized suspensions and clinical specimens.

From a methodological perspective, centrifugation is essential for concentrating *M. tuberculosis* due to its low specific gravity and slow natural sedimentation rate [10]. Insufficient centrifugal force may therefore contribute to reduced bacillary recovery, increasing the risk of false-negative results, particularly in paucibacillary disease. This may lead to delayed diagnosis and suboptimal treatment monitoring. Failure to detect non-responding patients by the second month of therapy may further contribute to relapse or the emergence of drug-resistant tuberculosis, which requires longer and costlier treatment regimens [18,19].

To assess the effect of centrifugation duration, specimens were processed at 3,000 × g for 20 and 40 min. Smear positivity remained unchanged between the two durations, with 80% positivity observed in both groups (95% CI: 55–100%; *p* = 1.000). In contrast, ZN smear grading increased from 1+ to 2+ with extended centrifugation time (*p* = 0.016), indicating improved smear intensity without a corresponding increase in detection rate.

Overall, these results suggest that optimizing relative centrifugal force may enhance the performance of ZN smear microscopy in clinical specimens, particularly in samples with heterogeneous bacillary distribution. However, extending centrifugation time appears to have limited impact on smear positivity.

Although WHO-recommended molecular assays such as GeneXpert MTB/RIF provide high diagnostic sensitivity, smear microscopy and culture remain essential for routine diagnosis and treatment monitoring in resource-limited settings [20]. Therefore, optimizing pre-analytical processing steps, including centrifugation parameters, may improve bacillary concentration and smear intensity in clinical sputum specimens.

A limitation of this study is the use of frozen and thawed clinical sputum specimens. Freeze–thaw cycles may alter sputum rheology and mucin structure, potentially affecting bacillary distribution and sedimentation behavior [10–12,14,16]. Although ZN microscopy detects acid-fast bacilli independent of viability, such changes may influence pellet formation efficiency compared with fresh specimens processed under routine conditions. A second limitation is the relatively small number of clinical specimens, which may limit generalizability.

## Conclusion

This study demonstrates that centrifugation conditions influence bacillary recovery as measured by ZN smear microscopy. In the laboratory *M. tuberculosis* H37Rv strain, increasing relative centrifugal force up to 3,000 × g did not significantly improve smear positivity, while higher force mainly affected smear grading without further gains in detection. In contrast, clinical sputum specimens showed a significant increase in ZN smear grading with increasing centrifugal force, although smear positivity remained unchanged across conditions.

Extension of centrifugation time from 20 to 40 min at 3,000 × g did not improve smear positivity in either *M. tuberculosis* H37Rv or clinical specimens, but resulted in improved smear grading in clinical samples only. These findings indicate that increasing centrifugal force may enhance bacillary concentration in clinical sputum, resulting in improved smear grading without a corresponding increase in detection rate.

Overall, optimization of centrifugation parameters may improve ZN smear microscopy performance in clinical sputum specimens under experimental conditions, particularly in samples with heterogeneous bacillary distribution. However, the study did not assess diagnostic accuracy against culture- or molecular-based reference standards; therefore, findings should be interpreted as improvements in smear performance rather than diagnostic sensitivity.

Further studies using fresh clinical specimens and reference-standard comparisons are required to determine the clinical impact of optimized centrifugation protocols in routine tuberculosis diagnosis.

## Supporting information

S1 File Data. (XLSX)
